# 2286: HOME Cell 2.0. Extending i2b2 to support community health outcome monitoring and evaluation via web-accessible software

**DOI:** 10.1017/cts.2017.65

**Published:** 2018-05-10

**Authors:** William G. Adams, Michael Mendis, Shiby Thomas, David Center, Sara Curran

## Abstract

OBJECTIVES/SPECIFIC AIMS: The primary objective of this effort is to develop and distribute an easy to use i2b2 component that is capable of evaluating diverse complex relationships for a wide variety of exposures and outcomes over time. In this manner we are able to leverage the unique design of the i2b2 database to support health services research, comparative effectiveness, and quality improvement using a single tool. Furthermore, our novel database redesign has the potential to provide user-friendly access to individual and group CHC data for CER. METHODS/STUDY POPULATION: For this project we used software experts, clinical informatics specialists, and the existing i2b2 open-source software to convert our legacy HOME Cell into a web-client version. The tool will be used to study health outcomes within a network of Boston based Community Health Centers and the largest safety-net hospital in New England, Boston Medical Center. RESULTS/ANTICIPATED RESULTS: The new web-client HOME Cell will allow i2b2 users to model virtually any exposure (including therapeutic interventions such as medications or tests) in i2b2 against any outcome accounting for complex temporal relationships and other factors. In addition we plan to use our new Community Health Center views to enhance our community engagement activities by allowing direct access to their data for our partners. DISCUSSION/SIGNIFICANCE OF IMPACT: Our project addresses multiple national priorities related to data sharing, clinical research informatics, and comparative effectiveness. The web-client version of the HOME Cell substantially improves our community’s access to HOME Cell functionality and is a novel, sharable resource for use within the CTSA/NCATS community. Our approach provides a new way to perform large-scale collaborative research without the need to actually move patient-level data and has demonstrated that CER, health services research, and quality measurement can share a common framework. In addition, and as demonstrated in our earlier pilot work, the HOME Cell also has the potential to support large-scale multivariate analyses in a distributed manner that does not require sharing of patient-level data. We believe our approach has great promise for supporting the reuse of clinical data for rapid, transparent, health outcome assessments on a national scale. Our efforts support multiple strategic goals including: (1) support for building national clinical and translational research capacity by enhancing a broadly adopted informatics tool (i2b2); (2) enhanced consortium-wide collaborations by offering a tool that can be easily shared within the CTSA network to support multi-institutional collaboration; and (3) improving the health of our communities by offering a tool that has the potential to provide new insights into health care processes and outcomes that could drive innovation and improvement activities.

